# Novel mobbing strategies of a fish population against a sessile annelid predator

**DOI:** 10.1038/srep33187

**Published:** 2016-09-12

**Authors:** Jose Lachat, Daniel Haag-Wackernagel

**Affiliations:** 1 University of Basel, Department of Biomedicine, Basel, CH-4056, Switzerland

## Abstract

When searching for food, foraging fishes expose themselves to hidden predators. The strategies that maximize the survival of foraging fishes are not well understood. Here, we describe a novel type of mobbing behaviour displayed by foraging *Scolopsis affinis.* The fish direct sharp water jets towards the hidden sessile annelid predator *Eunice aphroditois* (Bobbit worm). We recognized two different behavioural roles for mobbers (i.e., initiator and subsequent participants). The first individual to exhibit behaviour indicating the discovery of the Bobbit directed, absolutely and per time unit, more water jets than the subsequent individuals that joined the mobbing. We found evidence that the mobbing impacted the behaviour of the Bobbit, e.g., by inducing retraction. *S. affinis* individuals either mob alone or form mobbing groups. We speculate that this behaviour may provide social benefits for its conspecifics by securing foraging territories for *S. affinis*. Our results reveal a sophisticated and complex behavioural strategy to protect against a hidden predator.

Mobbing in the animal kingdom is described as an approach towards a potential predator followed by swoops or runs, sometimes involving a direct attack with physical contact by the mobber^1^. Mobbing is well characterized in birds, mammals, and even invertebrates^1^,^2^,^3^,^4^,^5^. Most of the reported mobbing behaviours initiated by a prey species involve directed harassment of a mobile predator^1^,^3^,^6^,^7^,^8^,^9^. Hidden, ambushing predators are a major threat to prey species. The timely discovery of a predator allows the danger zone to be avoided and enables countermeasures to be directed against the predator, including mobbing or even direct attacks^8^. In contrast to solitary living prey, social groups can profit from warning each other of ensuing dangers. Usually, mobbing occurs as a group phenomenon, but mobbing can also be performed by a single individual^3^,^10^,^11^.

*S. affinis* is a common demersal species that lives and feeds near the seabed of the western Pacific^12^. *S. affinis* preys on copepods, microcrustaceans, shrimps, and echinoderm larvae^12^,^13^. Juvenile *S. affinis* individuals are easily recognizable by the dusky-brown mid-lateral stripe on their sides. According to our field observations we suppose that immature *S. affinis* individuals forage in mobile groups that are restricted to a home range. Thus, it would be highly advantageous for these individuals to know to location of a hidden predator.

One such hidden ambusher of demersal species of fish is the Bobbit worm *Eunice aphroditois.* The Bobbit worm lives in the sedimentary beds of warmer oceans, including the Indo-Pacific and Atlantic^14^. The largest specimen of a recorded Bobbit worm was 299 cm long, weighing 433 g, and it may be the longest of the polychaete worms^14^. Bobbits reside in mucus-lined borrows in rocks or sediments. During the day, Bobbits are buried in the loose seabed waiting for a stimulus on one of their five antennae that protrude from the hidden burrow to lure prey fish (Fig. 1a). Armed with jaws with sharp teeth, Bobbits attack when they sense prey, grasping and dragging them down into their burrow (Fig. 1b). At night, the Bobbit changes its strategy and actively hunts by protruding from its burrow and snatching passing prey (Fig. 2 and Supplementary Movie 1). Bobbits have two eyes on the prostomium^15^ and five striped antennae that exhibit wormlike movement and presumably serve to lure and detect prey. Preliminary field experiments by one of the authors showed that predation by Bobbits can be triggered only by a lure that moves like a living fish (unpublished observation by J. Lachat). Additionally, during the night, the beam of a torch can trigger an attack of the Bobbit. Therefore, Bobbits appear to detect their prey mainly by visual and tactile cues. Bobbits are known to bite humans and can cause severe wounds^16^.

We performed underwater field observations by attracting *S. affinis* as a potential prey to the location of a Bobbit to study its behaviour towards this enemy. Remarkably, we discovered that the potential prey, *S. affinis*, initiates mobbing attacks towards the hidden Bobbit worm that in some cases eventually led to the Bobbit retracting into its burrow.

## Results

At our study site, we discovered four Bobbits in an area of 15,000 m^2^. Two Bobbits lived at a distance of 2.57 m apart. The other individuals were 16.9 and 25 m away from the former two individuals. The habitat use of *S. affinis* (frequency and length of stay in the area) was investigated by pursuing *S. affinis* individuals during the daytime. The individually pursued *S. affinis* commuted between the sand bottoms and the coral blocks (Fig. 3).

The sand bottoms were used mainly for searching for food. In the coral blocks, the pursued individuals searched for food and joined others with unclear interactions. Occasional jostling and chasing away could be observed. Eight individuals that were followed from behind during one dive used an area of approximately 80 × 100 m. During the night dives, several individuals were found resting in the coral block patches and on the sand bottom close to the coral blocks. Resting *S. affinis* individuals show a changed pattern with dark patches and a faded midlateral stripe (Fig. 4).

We analysed 54 mobbing encounters by *S. affinis*. Mobbing was performed exclusively by groups of immature *S. affinis* (54/54 observations). Mobbing behaviour was displayed either when a *S. affinis* individual discovered the hidden Bobbit worm (scenario 1, 51 observations) or after witnessing the predation of a conspecific (scenario 2, three cases). These two scenarios resulted in two different group dynamics of the mobbing behaviour.

When mobbing resulted after visual discovery of the Bobbit (scenario 1), the *S. affinis* individuals initiated a characteristic sequence of behaviours (Supplementary Movie 2).

**Figure 1:**
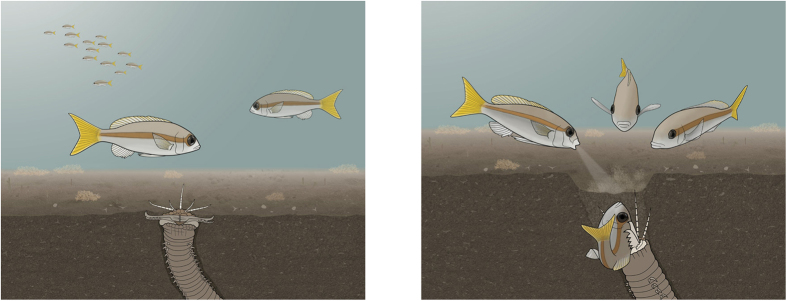
Predation of *Scolopsis affinis* during daytime by the Bobbit worm and subsequent mobbing. (**a**) The ambushing Bobbit is covered with sand and lures its prey with the protruding antennae; the jaws are under tension like an armed spring trap. (**b**) The Bobbit grasps and tears its prey into its burrow, and sand slips into the pit. Other *S. affinis* individuals approach and mob the Bobbit by blowing water jets into the pit.

**Figure 3:**
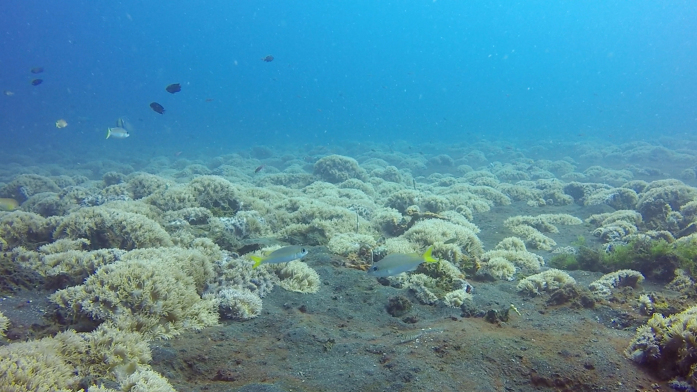
Habitat of *S. affinis* in the Lembeh Strait. *S. affinis* individuals commute between black sand bottom spots and low coral blocks. In the foreground, two immature *S. affinis* individuals swim over the sand bottom.

**Figure 4:**
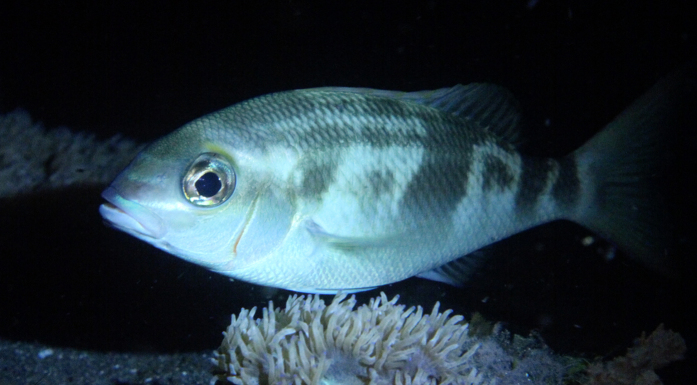
Resting *S. affinis*. During the night, *S. affinis* individuals lie close to coral blocks and show a changed pattern.

We analysed the visual cues that presumably triggered the subsequent mobbing (Table 1). A flat depression or a visible antenna presumably indicates an ambushing Bobbit. A strictly delimited burrow opening indicates that the Bobbit has retracted into its burrow (Fig. 1b). There was no correlation between the quality of the cue with the number of water jets directed towards the Bobbit.

**Figure 5:**
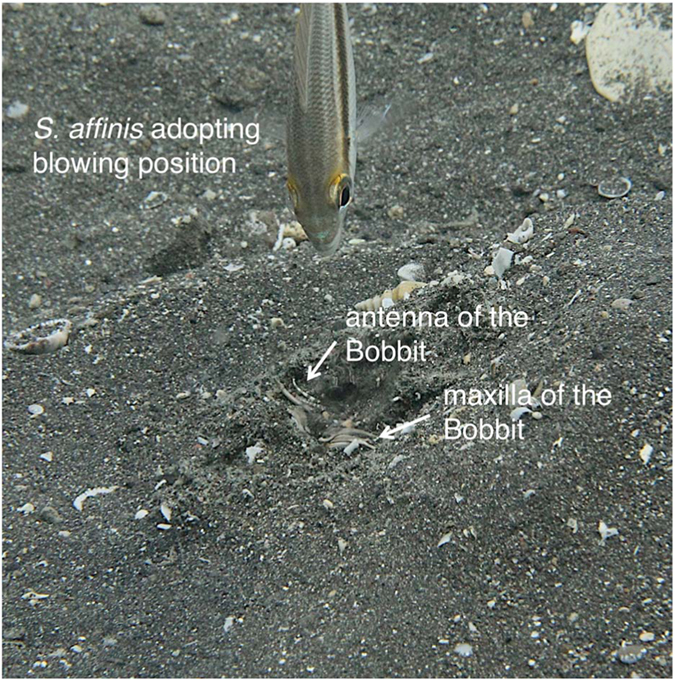
*S. affinis* in blowing position. The video still shows an experimental situation in which the head of Bobbit was exposed by the authors. An *S. affinis* individual directs a water jet towards the Bobbit.

We defined different behavioural roles of individuals of the mobbing group. The “initiator” is the first individual to exhibit behaviour indicating the discovery of the Bobbit. “Participants” are the subsequent individuals in chronological order that join the mobbing group. Typical mobbing behaviour is characterized as including a sequence of certain behavioural elements: (1) After initial discovery of the buried Bobbit worm, the initiator stops its foraging activity. (2) The initiator approaches the burrow, visually fixating on it by adopting an inclined posture. The approach is characterized by a series of movements towards the predator interrupted by stationary pauses and sometimes alternating with movements away from the predator. The majority of individuals that join the initiator also adopt an inclined posture with the head pointing towards the opening of the burrow. (3) A second approach is often followed by a more inclined posture almost reaching a vertical position. (4) When blowing, the individuals direct one or more water jets in the direction of the burrow, whirling up sand (Fig. 5). Mobbing is terminated when the involved individuals disband and swim away.

The number of mobbing individuals, including the initiator and the participants, varied from 1–7 (mean of 3.10, s.d. of 1.6, and median of 3 individuals, Table 2). The mobbing sequence (including predation cases) lasted 4–62 sec with a mean of 21.5 sec (s.d. of 11 sec, median of 20.5 sec). At any phase of the mobbing sequence, other *S. affinis* individuals would join the group. In 5 of 51 cases (9.8%), *S. affinis* only approached the Bobbit but did not blow. During the remaining 46 observations, *S. affinis* directed water jets at the Bobbit. In 4 cases, the Bobbit retracted at least one antenna, and in 15 cases, the Bobbit retracted completely into its burrow, made visible by sliding sand creating a crater (Supplementary Movie 3). In 35 cases, we could not identify a reaction in the videos. In 5 cases, an *S. affinis* individual mobbed alone without additional participants or observing individuals.

**Figure 6:**
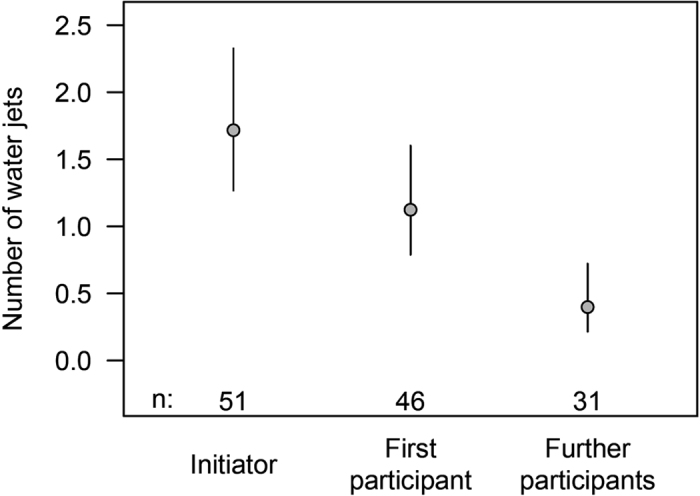
Earlier participants of the mobbing group displayed more mobbing behaviour through a higher amount of water jets. The average number (with a 95% Bayesian credible interval) of water jets directed towards the Bobbit worm by the first *S. affinis* (initiator) that discovered the worm was higher than the average number of water jets by the next joining individual (first participant) and the average number of water jets by the subsequent joining members of the mobbing group (later participants). Cases with predation are excluded.

**Figure 7:**
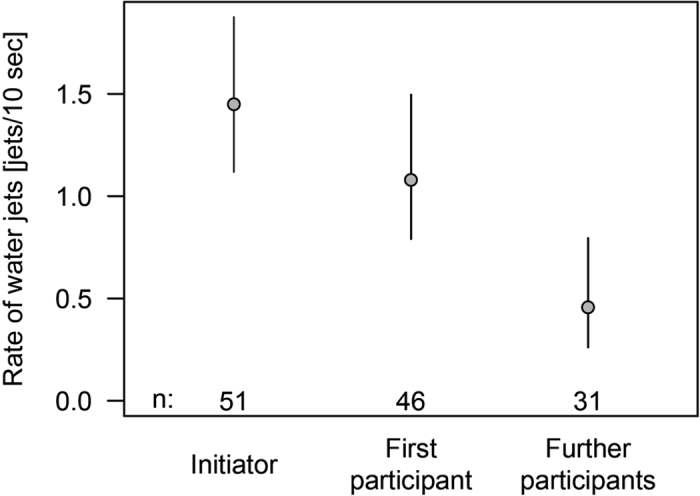
Earlier participants of the mobbing group also displayed more mobbing behaviour per unit of time. The average rate of water jets (number of jets per 10 sec, with a 95% Bayesian credible interval) against a Bobbit worm by the first *S. affinis* that discovered the worm (initiator) compared with the average rate by the next fish joining the mobbing (participant 1) and the average rate by the fishes joining afterwards (further participants).

The average number of water jets, as well as the average rate of water jets per unit of time, decreased along with the chronological position of the fish (Figs 6 and 7). The initiator emitted, on average, more water jets than subsequent joining participants (mean of 0.59 additional jets, 95% Bayesian credible interval −0.06–1.28 jets), and the first participant emitted significantly more jets than the subsequent joining participants (Fig. 6). The first participant produced, on average, 0.50 [CrI: 0.31–0.77 jets] more jets than the subsequent participant. The duration of mobbing tended to decrease from the initiator to the first participant (non-significant decrease by 1.42 [CrI: −0.98; 3.73] sec), and it decreased from the first to later participants (significant decrease by 4.02 [CrI: 1.27; 6.67] sec).

The rate of water jets per unit of time also was higher by the initiator (non-significantly higher by 0.37 [CrI: −0.14–0.87] jets per 10 sec compared with the first participant) and higher for the first participant compared with later participants (significantly higher by 0.51 [CrI: 0.30–0.76] jets per 10 s; Fig. 7). In none of the cases was *S. affinis* predated upon during or directly after the mobbing.

Next, we characterized the mobbing behaviour after predation (scenario 2). We analysed three video recordings of mobbing after predation (Supplementary Movie 4). The analysis revealed that in two of the three cases, the Bobbit snatched the *S. affinis* when it immersed into the sand directly above the opening of the burrow (Fig. 1), presumably attracted by food or the lure of an antenna. After it pulled its prey into the burrow, the Bobbit’s withdrawal caused pulsing movements of sand (Supplementary Movie 3). The entire process from the snatching of a prey until complete disappearance lasted approximately 0.1 s. Group members that witnessed the predation immediately approached the burrow and started the mobbing behaviour. This behaviour resulted in group dynamics clearly distinguishable from those in scenario 1 (mobbing after visual discovery) because there was no clear initiator of the mobbing behavior. The subsequent sequence of mobbing behaviour was similar to that described for mobbing after discovery. In two of the three mobbing events after predation, *S. affinis* individuals blew water jets at the submerged Bobbit worm. Due to the quick snatch and pull of the victim into the burrow, clouds of substrate were whirled up, unveiling food particles that were picked up by *S. affinis* and in one case by a wrasse (*Pteragogus* sp.) that had joined the mobbing group.

**Figure 8:**
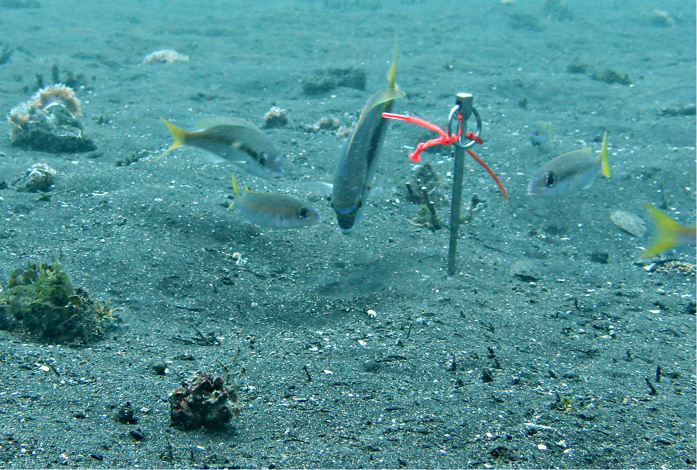
*Scolopsis monogramma* performing identical mobbing behaviour as *S. affinis.* Three *S. affinis* joined the *S. monogramma* in directing water jets towards the opening of the burrow of a Bobbit.

Fifty-one of 54 mobbing groups were monospecific. In 3 cases, two other fish species (*Pteragogus spec*., *Petroscirtes breviceps*) joined the mobbing group. These species approached and adopted the same posture as *S. affinis* but never exhibited water blowing. In 2016, we observed and documented a *Scolopsis monogramma* individual that had joined a group of *S. affinis* individuals (Fig. 8). The *S. monogramma* individual performed identical mobbing behaviour as described for *S. affinis* by directing 3 water jets in the direction of the burrow opening of a Bobbit (Supplementary Movie 6). The *S. affinis* individuals that were present approached, but they did not participate in blowing. Because we did not observe the beginning of the mobbing sequence, this observation was not included in the statistics.

Table 3 shows that in 16 observations (34.8%), the Bobbit showed a visible reaction by retracting an antenna or completely retracting into its burrow.

## Discussion

We only observed immature *S. affinis* individuals performing mobbing. Single adults could be seen only in a few cases, and they did not join the mobbing groups. There is a high possibility that adults live solitary and nocturnal lives, as described for *Scolopsis bilineatus*^17^, and therefore would not profit from this mobbing behaviour. The initiator directed more jets than the second joining fish (Participant 1), and this participant directed more jets than the following participants. The initiator is the first individual to discover the Bobbit and be aware of this direct danger. We suspect that the initiator is more aroused than the subsequent participants, which are made aware of the Bobbit by the behaviour of the initiator. Therefore, the initiator directs, absolutely and per unit of time, more water jets than the participants. Accordingly, participant 1 that has been “informed” about the Bobbit threat by the behaviour of the initiator appears to be under a higher arousal level than the subsequent participants and so on. This hypothesis is supported by the higher numbers of water jets emitted by participant 1 in comparison with the subsequent participants.

Several authors^1^,^2^,^3^,^4^,^6^,^10^,^18^,^19^ have analysed and discussed the costs and benefits of approaching and mobbing a predator. The potential costs that might be relevant for *S. affinis* when mobbing a Bobbit include an increased risk of mortality, lost opportunity costs and energetic costs. Blowing may confuse the predator, thereby potentially inhibiting its attack. Accordingly, we never observed a predation event during mobbing behaviour within the time frame of observation that started with the discovery by the initiator and ended with the departure of the last individual. Additionally, we observed in 16 of 46 observations direct effects of the mobbing on the Bobbit, which reacted by the retraction of antennae or complete withdrawal. Bobbits are ambushers, and they attack their prey when they can surprise it. Therefore, we speculate that the risk of mortality during mobbing is low. The complete mobbing events lasted 21.5 sec, on average; hence, we speculate that the energetic costs and lost opportunity costs of foraging are relatively low. In other fish species, the presence of shoal mates may increase the probability of detecting predators, reduce the chance of being predated, and confuse predators, and the increased information about the presence and location of predators may benefit other members of the shoal^19^. There is direct experimental evidence that fathead minnows (*Pimephales promelas*) learn to recognize predators and dangerous locations when they witness the response of experienced conspecifics to chemical stimuli from predators or dangerous sites^20^. This could also be true for *S. affinis*. In particular, younger individuals sometimes approached the mobbing closely to inspect the situation (Supplementary Movie 5). The mobbing, therefore, also appears to have an educational effect on informing other individuals about the presence and location of the predator. We suggest that one of the benefits of forming a foraging group is the communication about the location of Bobbits because many eyes see more than only two eyes. We assume based on our field observations that S affinis shows a certain degree of site fidelity and can profit from the knowledge of Bobbit locations. Further research should include other places where Bobbits and *S. affinis* live to determine whether the documented mobbing is an isolated phenomenon or a widespread behaviour of *S. affinis*.

We documented an adult *Scolopsis monogramma* in the company of a group of four *S. affinis* individuals that performed an identical mobbing behaviour as *S. affinis*. This astonishing observation may indicate that mobbing by directing water jets to an ambushing enemy is more widespread. Further research is necessary to clarify the relevance of this observation and the possibility of other related species living in the same habitat that exhibit mobbing behaviour against Bobbits.

## Methods

### Field observations

Underwater observations were conducted from 2012–2016 at the dive spot Air Prang (Lembeh Strait, Sulawesi, Indonesia, N 1°28′04.82″E125°14′05.52″). The study area has a black sand bottom with an inclination of approximately 6° with scattered patches of low coral blocks. In total, 90 dives at depths of 12–15 m were performed with the assistance of a dive guide. The depth and distance between the Bobbits were recorded using a dive computer and a pocket rule. The positions of the Bobbits were reconnoitred and marked during the night. By day, the spots were visited again where the experiments were performed. Sting rays (*Dasyatis kuhlii* and *Taeniura lymma*), which are widely distributed at the Lembeh Strait, are followed by foraging *S. affinis*. When the rays whirl up sand when digging for food by flipping their wings, *Scolopsis* individuals approach and pick up small food particles. By whirling up sand by hand, we copied this behaviour to lure *S. affinis* to the Bobbits.

During 8 dives, we pursued individual *S. affinis* by swimming behind at a distance of approximately 3–7 m for an average of 1 hr. When the pursued individual approached us, we stopped following. All positions were indicated on an underwater notebook and later transferred to a map of the study site. The pursuit had to be relinquished in 3 cases after 47, 42 and 16 min when the individual was lost. During 3 night dives, we searched for *S. affinis* in the same area where they were observed during the day.

The observations were videotaped with a Canon 1Dc Ultra-HDTV video camera protected by an underwater housing fixed on a tripod. We only used complete recordings that included the start and end of the mobbing. The start of the mobbing was defined as the approach, and the end was defined as the swimming away of the last individual. The scenes were analysed by both authors individually with frame-by-frame analysis if necessary. In the case of different results, the sequence was re-analysed. We recorded the following behavioural components: (1) visual cues that presumably initiated mobbing (sight of the Bobbit and its burrow, the witnessing of a predation), (2) the number of individuals attending the mobbing, (3) the duration of the mobbing sequence, (4) the time the individuals attended the mobbing, (5) the number of water jets directed against the Bobbit by the initiator (individual that discovered the Bobbit) and participants (individuals that joined the mobbing), (6) other participating fish species, (7) the behavioural response of the Bobbit and (8) other activities of *S. affinis* during mobbing, e.g., feeding. The methods and the experimental protocols were approved by the Mutual Animal Experimentation Committee of the Swiss Cantons Basel-Town, Basel-Country and Argovie.

### Statistical analysis

To model the number of water jets per behavioural type (initiator, participant 1, further participants), we built a Poisson mixed model with the behavioural type as the fixed factor and the mobbing group as the random factor and an observation-level random factor to account for over-dispersion. To obtain the mean rates, we used the same model but added the duration of the mobbing as an offset. The mean duration of mobbing was modelled using a normal linear mixed model with the type of behaviour as the fixed factor and the mobbing group as the random factor. Ninety-five per cent Bayesian credible intervals were based on 10,000 samples from the posterior distribution and assuming flat priors.

## Additional Information

**How to cite this article**: Lachat, J. and Haag-Wackernagel, D. Novel mobbing strategies of a fish population against a sessile annelid predator. *Sci. Rep.* **6**, 33187; doi: 10.1038/srep33187 (2016).

## Supplementary Material

Supplementary Information

Supplementary Movie S1

Supplementary Movie S2

Supplementary Movie S3

Supplementary Movie S4

Supplementary Movie S5

Supplementary Movie S6

## Figures and Tables

**Figure 2:**
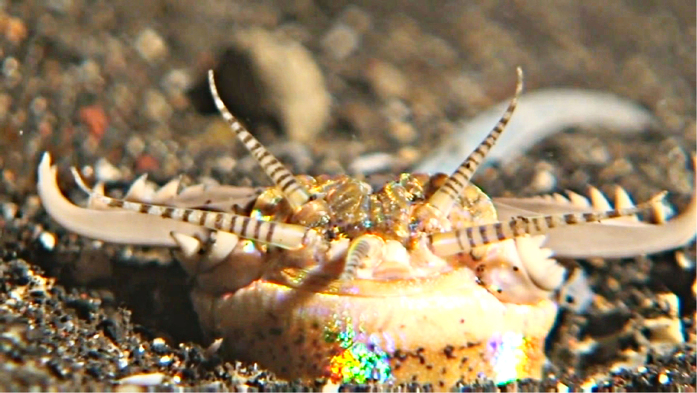
Head of a Bobbit, *Eunice aphroditois.* The video still shows an actively hunting Bobbit at night. With its jaws, the Bobbit attempts to snatch passing prey perceived by the antennae.

**Table 1:** Visual cues that presumably initiated mobbing.

Visual cue	Observation	Percentage
No cue visible	1	1.9%
Flat depression	18	33.3%
Visible antenna	10	18.5%
Burrow opening	22	40.7%
Predation	3	5.5%
Total	54	99.9%

**Table 2:** Number of mobbing *S. affinis.*

Number of mobbing individuals	Observations	Percentage
Alone	5	9.8%
Group of 2	16	31.4%
Group of 3	16	31.4%
Group of 4	7	13.7%
Group of 5	3	5.9%
Group of 6	2	3.9%
Group of 7	2	3.9%
Total	51	100%

**Table 3:** Consequences of water jets directed at Bobbit.

Visible reaction to water jets	Observations	Percentage
No reaction	30	65.2%
Retraction of antenna	4	8.7%
Complete retraction	12	26.1%
Total	46	100%
