# Supplementary material for: Novel mobbing strategies of a fish population against a sessile annelid predator
Source: Sci Rep. 2016 Sep 12;6:33187. doi: 10.1038/srep33187 (PMC5018815; doi:10.1038/srep33187)
Supplement: Supplementary Information [file srep33187-s1.doc]

**Novel mobbing strategies of a fish population against a sessile annelid predator**

Jose Lachat & Daniel Haag-Wackernagel

**Supplementary Movies**

**Supplementary Movie 1: Hunting behaviour of a Bobbit *(Eunice aphroditois*) at night.**

The video starts with the ambushing Bobbit. In contrast to daytime hunting, the head and the first few body segments protrude out of the burrow. When the prey fish *(Apogon moluccensis)* passes by, the Bobbit quickly captures it and withdraws into the burrow.

**Supplementary Movie 2: Discovery of a Bobbit by a foraging *Scolopsis affinis* with subsequent mobbing.**

While foraging in a group, a small *S. affinis* (the initiator) accidentally discovers a Bobbit. Using a strong fin flap, *S. affinis* escapes the danger zone and adopts a horizontal position while observing the Bobbit. Then, the initiator adopts an inclined position pointing to the burrow and blows a sequence of water jets directed at the Bobbit. Other *S. affinis* join the initiator (participators) by approaching. A second *S. affinis* approaches and starts blowing. At the end of the video, the initiator again directs one water jet at the Bobbit.

**Supplementary Movie 3: Water jets cause withdrawal of the Bobbit into its burrow.**

The video shows the effect of the mobbing. After the fish direct a series of water jets towards the Bobbit, the Bobbit withdraws into its burrow, visible due to sand gliding back to the burrow. Two *S. affinis* peck up food particles, presumably aware that the Bobbit is no longer a menace.

**Supplementary Movie 4: Predation of an individual *S. affinis* during the day.**

An individual *S. affinis* is grasped by a Bobbit and pulled into its burrow, visible from the sand swirled upward. Upon withdrawal into the burrow, sand shifts down. Three *S. affinis* approach and adopt a horizontal mobbing position while observing the burrow.

**Supplementary Movie 5: A small *S. affinis* is attracted to a mobbing group and observes the scene from nearby.**

The initiator discovers the Bobbit and starts mobbing by approaching and starting to blow water jets towards the burrow. This behaviour attracts others group members (participators), which join the initiator. A young *S. affinis* joins the mobbing group and presses forward to inspect the situation.

**Supplementary Movie 6: Mobbing by a *Scolopsis monogramma* in a group of *S. affinis.***

The movie shows the end of a mobbing sequence. The *S. monogramma* directs three water jets to the burrow opening of a Bobbit. Four *S. affinis* approach but do not participate in directing water jets.
